# The impact of emotion regulation strategies on experiential and physiological responses to negative emotional videos

**DOI:** 10.1098/rstb.2025.0138

**Published:** 2026-09-24

**Authors:** Wilson P. H. Lim, Hannah S. Savage, Beth F. Longley, Hugo D. Critchley, Tim Dalgleish, Camilla L. Nord, Sarah N. Garfinkel

**Affiliations:** ^1^ Institute of Cognitive Neuroscience, University College London, Alexandra House, 17–19 Queen Square, London WC1N 3AZ, UK; ^2^ Department of Clinical Neuroscience, Brighton and Sussex Medical School, University of Sussex, Falmer, Brighton, East Sussex BN1 9PX, UK; ^3^ MRC Cognition and Brain Sciences Unit, University of Cambridge, 15 Chaucer Road, Cambridge CB2 7EF, UK; ^4^ Department of Psychology, University of Cambridge, Downing Street, Cambridge CB2 3EB, UK; ^5^ Department of Psychiatry, University of Cambridge, Herchel Smith Building, Forvie Site, Robinson Way, Cambridge CB2 0SZ, UK

**Keywords:** emotion, emotion regulation, autonomic nervous system, self-distancing, self-immersing, affective induction

## Abstract

Emotion regulation strategies influence how individuals experience and express emotions. Previous work suggests that self-distancing can reduce negative emotional responses, whereas self-immersion can amplify them. This study quantified how these emotion regulation strategies, applied during exposure to negative and neutral videos, shaped self-rated emotional experience across valence, arousal and intensity dimensions, influenced the free production of emotion-descriptive labels and altered measures of respiratory and cardiovascular physiology. A total of 45 participants took part in a task during which they were prompted to simply watch, or to self-distance from or self-immerse in, the emotional content of negative or neutral videos, which were presented in 12, 30-s blocks. Their rated emotional experience (valence-arousal-intensity ratings and free emotion labels) was recorded, along with cardiovascular and respiratory activity. Overall, self-distancing and self-immersing respectively attenuated and amplified aspects of subjective emotion experience relative to comparator conditions as predicted. Self-distancing reduced freely generated emotion labels corresponding to fear, but had less impact on labels corresponding to disgust, sadness and anger. The effects of emotional regulation strategies on cardiorespiratory physiology (e.g. heart rate) potentially also differ between individuals. Together, this work validates a framework for dissecting components of induced emotion, highlighting commonalities and variability in experiential and physiological reactivity and effects of regulation.

This article is part of the theme issue ‘Mechanisms, development, phylogeny and functions of emotional expressions’.

## 1. Introduction

Emotions are evoked psychophysiological and behavioural states, characterized by altered experiential feelings, reorientation of attentional and motivational resources, with shifts in action tendencies and behaviour that are accompanied by changes in internal physiology [1–4]. Influential theories of emotion propose that these changes in bodily physiology, mediated involuntarily in part by the (patterned) autonomic nervous system, facilitate adaptive behaviour (e.g. by increasing cardiac output and ventilation to prepare and support fear-related escape) and, through interoceptive representation, underpin subjective affective and emotional feelings. Such feelings are further shaped and categorized through cognitive appraisal (of perceived triggers and social/environmental contexts).

The James–Lange theory contributed to this model by arguing that emotions can be reduced to their physiological signatures [5,6]. Following Cannon [7], Schachter and Singer's two-stage model [8] emphasized the role of cognitive appraisals in driving discrete emotion experiences grounded in poorly differentiated bodily physiology [8–11]. The theory of constructed emotion [12] extends this logic to emphasize emotions as mind–brain–body phenomena, wherein interoceptive signals concerning bodily physiology are integrated with sensory (exteroceptive) and mnemonic information in the service of allostasis [12–15]. By contrast, the automaticity of homeostatic reflexive control of internal physiological changes, allostasis describes the more efficient, predictive control of health in which autonomic responses and behaviours are coordinated to mitigate anticipated threats to physiological health. Emotions represent the extension of allostatic control to motivational and social behavioural repertoires, supported by emotional feelings that are shaped by higher-order cognitive processes, such as appraisal and attribution [16,17]. This influence allows emotions to be subject to a degree of volitional control.

Emotion regulation refers to active, conscious or unconscious modification of the intensity, duration or nature of emotional experience and emotional responses, typically through adjusting (increasing or decreasing) the psychological and cognitive representation of positive and negative emotions, by altering behavioural expression, or through modifying the situation to change its emotional impact [18–20]. Emotion regulation can lead to changes in the specific types of emotions that people may experience, when and how these emotions are experienced, and how these emotions are expressed [21].

One emotion regulation strategy is *self-distancing* (or *distancing*), which is sometimes discussed under the broader construct of reappraisal [22–24] or as ‘decentering’ in a mindfulness context [25,26]. Self-distancing involves viewing negative or positive thoughts, memories or events in the world ‘at a distance’—for example, from the perspective of a detached or neutral observer [26]—to disengage from proximate inner experiences in order to allow negative perceptions and cognitions to be reinterpreted within a broader context [26–28]. Self-distancing forms a core component of interventions within cognitive behavioural therapy and mindfulness-based cognitive therapy. Correspondingly, when individuals are instructed to engage in self-distancing, negative emotional experiences are quantifiably reduced [27,29,30]. The effectiveness of self-distancing in regulating emotions is often contrasted with a *self-immersed* perspective, where individuals enhance the personal mental proximity to their emotional experience [31,32].

Emotion regulation is mostly applied to control and attenuate (usually negative) emotional experience, yet there are instances where it can be adaptive to enhance emotional experience, for example, in the treatment of anhedonia, which is characterized by emotional numbness [33]. *Self-immersion* (or *immersing*) increases the intensity of emotional responses [30], such that individuals who show more self-immersion exhibit intense emotional reactions [32,34–36].

However, further research is needed to understand how different emotion regulation strategies influence dissociable categorical (e.g. surprise, fear, disgust, etc.) and dimensional aspects of emotion (including valence, arousal and intensity). There is also a need to characterize more fully the physiological mechanisms engaged during emotion regulation, given the integrated contribution of internal physiological changes and their interoceptive representation to emotional responses and feeling states that reflect their allostatic underpinnings.

Relevant to this is the expression of cardiorespiratory coupling, as reflected in phenomena such as respiratory sinus arrhythmia (RSA), which typically dominates measures of heart rate variability (HRV). Measures of RSA capture how heart period shortens during inhalation and lengthens during expiration, through the influence of respiratory control on the brainstem (homeostatic) baroreflex, mediated by parasympathetic (vagus nerve) effects on the cardiac pacemaker. RSA and HRV reflect baroreflex function and its tuning by ‘top-down’ (allostatic) control that prioritizes the stress response over minute-to-minute blood pressure stability: suppression of the baroreflex allows heart rate (HR) and blood pressure to rise together in support of current or anticipated action-related behaviour. This parasympathetic-to-sympathetic shift in cardiovascular control is characteristic of many ‘negative’ emotional (fight-or-flight) states, evident as decreased RSA and (high-frequency or total) HRV power. By contrast, positive emotional and low-stress states are typically associated with a longer heart period (lower HR) and increased RSA/HRV. Nevertheless, these effects are often inconsistent with variable HRV changes reported during negative affective states [37,38] and, when including respiratory indices, heterogeneous patterns observed during emotions like sadness [10].

Measures of cardiorespiratory physiology nevertheless remain important in gaining a mechanistic understanding of emotion regulation: cardiorespiratory coupling ensures that autonomic control of cardiovascular arousal is dependent on respiratory function, which itself is more directly amenable to volitional control. Slower, deeper breathing will enhances RSA/HRV, thereby reducing putative physiological correlates (and associated interoceptive representations) of stress and unwanted negative emotions. In the present study, we therefore included detailed measures of respiratory function (including respiratory rate variability (RRV) and the ratio of inspiration to expiration time). Such respiratory measures receive comparatively little attention [39] yet complement cardiovascular measures (e.g. HRV) to reflect the autonomic (homeostatic/allostatic) regulation of bodily physiology [40,41] and provide more mechanistic insight into the physiological dimensions of emotional regulation [42].

We employed an emotional reactivity and regulation paradigm, with concurrent cardiovascular and respiratory recordings. The primary aims of our study were to (i) characterize self-reported emotional experience and physiological reactivity in response to negative emotional stimuli, relative to neutral stimuli, and (ii) quantify the impact of emotion regulation strategies designed to attenuate and augment emotional reactivity both on (i) aspects of subjective emotional experience, including freely-generated emotion labels and (ii) cardiorespiratory physiology.

To establish the construct validity of our stimuli based on participants’ ratings, we first aimed to ascertain whether our negatively valenced videos would be rated as more negative in valence and as more arousing and intense than neutral videos. We also hypothesized that intentional mental immersion in the emotional content of these videos would amplify ratings of negative valence, emotional arousal and intensity, when compared with the watching and distancing conditions. Similarly, we predicted that self-distancing from emotional content would cause the videos to be rated as having a less negative valence, and as less arousing and intense, when compared with both the watching and immersing conditions. We also expected that emotion labels generated after watching negative and neutral videos would show similar valence–arousal–intensity distributions, and we undertook exploratory analyses to test whether emotion regulation (immersion/distancing) strategy (relative to the watching condition) elicited distinct shifts in valence, arousal and intensity ratings for different emotions. We anticipated that physiological changes would mirror self-reported emotion measures, with quantifiable differences in physiological signatures of cardiorespiratory arousal (e.g. lower RSA/HRV, higher HR and respiratory rate) when viewing negative stimuli compared with neutral stimuli. Moreover, we predicted that, relative to watching, emotion regulation through distancing would attenuate physiological indices of cardiorespiratory arousal, whereas immersion would amplify cardiorespiratory arousal. Together, this allows us to determine a profile of autonomic involvement in emotional regulation processes.

## 2. Method

### (a) Participants

The study was approved by the University College London Human Research Ethics Committee (ICN-VW-01-08-2024) and participants were recruited through the University College London SONA recruitment platform and from the general population, via posters, online forums and word-of-mouth. Volunteers were first screened via an online screening form, and if eligible for the study, were invited to take part. Participants were excluded if they: (i) were not aged between 18 and 65 years; (ii) had a current diagnosis of diabetes, epilepsy, pain disorder, substance use disorder or a previous/current diagnosis of schizophrenia or bipolar disorder; (iii) had undergone previous cardiovascular surgery; (iv) had a cardiac pacemaker or implantable cardioverter-defibrillator; (v) had bradycardia; (vi) had a previous or current diagnosis of a significant cardiovascular or respiratory condition; or (vii) were currently taking antipsychotics, corticosteroids, opiates or antidepressants (specifically noradrenergic and specific serotonic antidepressants, tricyclic antidepressants, serotonin antagonist and reuptake inhibitors or monoamine oxidase inhibitors—selective serotonin reuptake inhibitors (SSRIs)/serotonin–norepinephrine reuptake inhibitors were not an exclusion criterion if dosage had not changed in the past month). We wanted our sample to be representative of affective range across the general population, and thus took an inclusive approach to affective symptoms and affective disorders. As such, we included participants with depression, generalized anxiety disorder, autism spectrum disorder (ASD), obsessive−compulsive disorder (OCD) and attention deficit hyperactivity disorder (ADHD) in order to examine a more naturalistic sample that reflects the larger population. All participants gave written informed consent, following a complete description of the study protocol. Participants received either course credits or monetary compensation for their participation.

A total of 51 participants were recruited. Six participants’ data were later excluded owing to missing values (opting out of the task, technical issues with the recording). The final sample included in analyses comprised 45 participants (12 male, 33 female) aged between 18 and 35 years (*M*_age_ = 22.22, *SD*_age_ = 4.23). Of these, two participants self-reported a diagnosis of ADHD, two a diagnosis of generalized anxiety disorder, two a diagnosis of ASD, one a diagnosis of OCD and one a diagnosis of depression. None of the participants reported taking cardiac medication, hypertensive medication or medication to treat respiratory conditions. One participant reported taking antidepressant medication (SSRI), but as per the inclusion/exclusion criteria, their dosage had remained stable for the previous month before their testing session. A priori simulation-based power analyses [43] were conducted, with model parameters (fixed effects, random effects and residual variance) informed by pilot data. Power was estimated via Monte Carlo simulations (*n* = 1000), in which models matching the planned analysis structures were iteratively refitted across varying sample sizes. Power analyses indicated that a sample size of 45 was sufficient to achieve adequate power (approx. 80%) to detect the effects of interest in the linear mixed-effects models used in the main analyses.

### (b) Material and measures

#### (i) Stimuli

A total of 26 neutral and 23 negatively valenced videos were selected from three previously validated datasets: Database of Emotional Videos from Ottawa [44], Open Library for Affective Videos [45] and the Cowen–Keltner dataset [46]. For videos in the Cowen–Keltner dataset that were included, the original videos with sound were sourced. Emotionally neutral videos depicted a variety of neutral scenes (e.g. a person washing a cup, a man jogging with his dog), while negative videos depicted a variety of unpleasant and emotionally arousing scenes (e.g. animal attacks and interpersonal violence). The short videos for each condition were selected such that they had broadly equivalent levels of viewer-rated emotional arousal and valence, as reported in the respective validation publications or datasets (see the supplementary material for more information about videos). Following selection of the videos, they were combined into 12, 30-s blocks (six neutral, six negative videos), with neutral and negative blocks matched for semantic content and mean emotional arousal and valence matched within each emotion regulation condition. Each block contained an average of four videos (min = 3 and max = 6) that were played one after another in succession. Three additional videos (one negative and two neutral) were selected for use in the training blocks. The task code was Python-based and run in PsychoPy (v.2023.1.3) [47] on a desktop PC with Windows 11.

#### (ii) Physiological data collection

Monitoring of HR and breathing pattern was achieved non-invasively using electrocardiography (ECG) and respiratory belts. ECG was recorded using three standard disposable pre-gelled Ag/AgCl electrodes (Ambu WhiteSensor, Denmark) placed in a modified 3-lead configuration, with two electrodes placed under the left and right infraclavicular fossa (just below the clavicle) and the ground electrode placed on the participant's lower left back. Two respiratory belts (Pneumotrac II, UFI, USA) were used to measure respiratory-related movement of the chest and abdomen related to thoracic and diaphragmatic breathing. The former encircled the chest just below the armpits, and the latter was placed just below the sternum. ECG and respiratory signals were sampled at 1000 Hz and were registered and amplified through the CED 1902 isolated pre-amplifier and CED Power1401-3A and recorded using the Spike2 software (v. 10.13; Cambridge Instruments, Cambridge, UK; see https://ced.co.uk/) in real-time on a laptop running Windows 11.

### (c) Procedure

The study was conducted in a quiet room kept at a comfortable temperature (21–23°C). After informed consent and basic demographic information were obtained, the ECG electrodes and breathing belts were placed on the participant. The current task was performed with a larger battery of tasks and questionnaires within a 2.5-hour testing session.

The instructions for the task were first explained to the participant, which included the different emotion regulation conditions: watch, distance and immerse. In the ‘watch’ condition, participants were told to sit and watch the videos. In the ‘distance’ condition, participants were told to engage in self-distancing, where they were instructed to take a mental ‘step back’ from their emotional reaction to the videos and to view the videos from a broader, calmer and less emotional perspective. It was also suggested to participants that one way of doing this was to imagine themselves as an external observer watching themselves watch the videos from a distance. In the ‘immerse’ condition, participants were told to watch the videos and imagine themselves, a loved one or a close friend in the situation shown in the videos. Participants were also instructed to try to engage fully with the content in the videos and, where possible, relate it to something that had happened to them or to someone they know. For example, participants could imagine a loved one as the subject of the video, and engage with the video content by relating the situation to their loved one (e.g. mourning the death of a loved one). Participants then completed a short quiz to ensure that they understood the instructions for the different emotion regulation conditions. If a participant answered any questions incorrectly, the instructions were repeated and the quiz was retaken. Following successful completion of the quiz, participants took part in three training blocks to ensure that they were familiar with the task and comfortable with viewing the mildly distressing content, before commencing the main section of the study.

The main study comprised 12, 30-s blocks, comprising videos that alternated between neutral and negative blocks. The emotion regulation condition was randomized between participants, who were randomly assigned to one of two task versions. In both versions, participants were first assigned to the watch condition for the first two blocks. Based on the task version they were assigned, this was followed by either two blocks of distance or two blocks of immerse, and finally by two blocks of the remaining (unexperienced) condition (immerse/distance). This sequence was then repeated for the final six video blocks. At the start of each block, participants were shown a screen for 2 s with instructions to relax, followed by a screen showing them the emotion regulation condition (watch/distance/immerse) for the upcoming block, which lasted 10 s. The 30 s video block was then played on screen.

After the end of each block, participants were asked to indicate how they felt (‘What did you feel?’) by placing a dot on an *affect grid*. This grid comprised two dimensions: the horizontal *x*-axis represented valence, while the vertical *y*-axis represented arousal. Following this, participants rated the intensity of their emotion by adjusting the size of the dot: the larger the dot size, the more intense the emotion experienced. Valence, arousal and intensity were scored on scales from 0 to 100. Participants were then asked to type up to five words to describe how they felt. Finally, participants were asked to rate how well they think they managed to view the videos according to the instruction given to them (see figure S1 in the supplementary material for a task overview). The minimum time between the end of the previous block and the beginning of the next was set to 15 s; if participants took less than 15 s to complete the ratings and labels, the relax screen for the following block was extended until the inter-block time reached 15 s. At the end of the study, participants were debriefed, thanked for their participation and compensated for their time.

### (d) Data processing and analyses

#### (i) Emotion labels

Emotion labels were first checked and corrected for spelling errors before the root word for each label (e.g. disgust: captures disgusting, disgusted) was extracted using a stemming algorithm. This was done using the *nltk* Python package via the *PorterStemmer* function [48]. After running the stemming algorithm on all freely generated labels, the top 10 most frequent root labels for negative and neutral video blocks were extracted. However, labels that did not meet a frequency of three or more within each of the emotion regulation conditions were not considered. The labels were then combined if they were semantically similar to each other to increase power (see supplementary material table S1 for a breakdown of label groupings and the full list of labels). These were carried out in order to mitigate limitations in subsequent statistical analyses (see §2d(iii)) that arise when labels occur at very low frequencies, as this can produce unstable estimates and may affect the reliability of downstream analyses. From the top 10 most frequent root labels, each for negative and neutral video blocks, the five most frequent negative root words (disgust, sadness, fear, anxiety and anger) and the three most frequent neutral root words (calm, happy and neutral) were obtained after combining semantically similar labels. This approach of combining labels was guided by conceptual overlap in affective meaning rather than fine-grained distinctions, and as such, the final categories reflect broader emotion families rather than highly specific emotional states.

#### (ii) Physiological data

Pre-processing for the collected physiological data was carried out in Python using the NeuroKit2 package [49]. ECG R-waves were identified to index the timing of each heartbeat and derive measures of HR and RSA/HRV: recorded ECG data were first cleaned by applying a 0.5 Hz high-pass Butterworth filter (order = 5), followed by a 50 Hz power line notch filter. R-peaks were identified using the modified EngZee algorithm by combining slope-based detection with adaptive thresholding, followed by peak detection once the QRS complex had been identified [50]. If detected, artefacts, such as ectopic beats, were corrected using an automatic algorithm that first detects abnormal values from the distribution of consecutive RR-intervals in the signal. These are then replaced with interpolated values [51]. Our threshold required the exclusion of recordings in which more than 1% of RR-intervals required correction; however, all recordings were included as they remained below this threshold. All data were visually inspected for missing or incorrect R-peaks, with manual addition or removal, respectively, if required.

The raw respiration data from both respiratory belts, reflecting dynamic changes in the circumference of chest and abdomen, were first cleaned by applying a 0.1–0.35 Hz bandpass Butterworth filter (order = 2). This was followed by constant detrending to remove any residual constant offset (e.g. from baseline shifts in respiration belts from belt tension or chest movement, residual DC offset, etc.) in order to centre the respiration signal around zero. Exhalation and inhalation onsets were detected using the method described in the BioSPPy Python package, by detecting zero-crossings in the recording where the cleaned signal crosses zero [52]. All data were visually inspected for exhalation and inhalation onsets, which were then manually corrected if onsets were incorrectly identified. Since exhalation and inhalation onsets from both the diaphragmic and thoracic breathing belts were almost perfectly correlated, only the data from the diaphragmic breathing belt were analysed.

Following data pre-processing, HRV and RRV indices were extracted using NeuroKit2 (see [49] for processing steps); RSA was calculated using the Porges–Bohrer method as specified in NeuroKit2. The included indices comprised the time-dependent measure of root mean square of successive differences (RMSSD) and frequency-dependent measure of high-frequency spectral power (HF; 0.15–0.40 Hz) for both HRV and RRV, RSA, mean HR, mean respiration rate and the ratio of inspiration to expiration time (I/E ratio = inspiration time/expiration time). The I/E ratio was calculated for every respiratory cycle and averaged across each block. Inspiratory duration was measured from the onset to the end of inspiration, whereas expiratory duration extended from the end of inspiration to the onset of the subsequent breath, thus incorporating both inspiratory and expiratory pauses.

#### (iii) Statistical analysis

Statistical analyses were conducted using R v.4.4.3 in RStudio [53,54]. Self-reported emotional ratings (valence, arousal and intensity) and derived physiological measures were analysed using linear mixed-effects models (LMMs; *nlme* package [55]). To verify that participants perceived the negatively valenced videos as intended, we confirmed whether negatively valenced videos would be rated as being more negatively valenced. Separate models tested the effects of video type (neutral versus negative) and emotion regulation condition, with task version (order of emotion regulation conditions) as a covariate. Sex was initially included as a covariate owing to known gender differences in autonomic reactivity and in emotion regulation [56–59]; however, its inclusion did not significantly improve model fit nor alter the pattern of results. As such, the more parsimonious models without sex were carried out and reported. Medication and psychiatric status were also included as covariates in the models. Random intercepts were also specified for each participant to account for within-participant correlations. To account for potential heteroscedasticity across video types and conditions, variance structures allowing different residual variances by video type or condition were specified, respectively. Corrections for multiple comparisons were done across LMMs using the Benjamini–Hochberg procedure, while significant main effects were followed up with pairwise comparisons using estimated marginal means (*emmeans* package [60]) with Tukey's correction. Additional LMMs were run to examine the interaction effects of video type and emotion regulation condition on self-reported emotional ratings and derived physiological measures. Where significant interactions were found, additional LMMs were run to examine the effects of condition separately within negative and neutral video types, similarly correcting for multiple comparisons across models using the Benjamini–Hochberg procedure, with significant main effects being followed up with Tukey-corrected pairwise comparisons.

In addition, given that negative emotions can both increase and decrease HR [10], absolute change scores were calculated to determine the impact of emotion regulation on cardiac reactivity, irrespective of the direction of change. One-sample *t*-tests were carried out on the absolute difference scores in mean HR to evaluate whether emotion regulation (distance/immerse) produced any deviation from the baseline watch condition. These tests were conducted across all videos and separately for each condition comparison (distance versus watch and immerse versus watch), and adjusted for multiple comparisons using the Benjamini–Hochberg procedure. Following that, an LMM was run to examine the interaction effects of video type and condition comparison, and one-sample *t-*tests were conducted separately for negative and neutral videos, with multiple comparisons corrections applied in the same manner.

Frequencies of labels between label groups were compared using Friedman tests, and where significant, pairwise comparisons were carried out using Wilcoxon signed-rank tests, correcting for multiple comparisons using the Benjamini–Hochberg procedure. To visualize and examine how the different label groups were distributed on the affect grid, within each label group, each emotion label was plotted according to its corresponding self-report ratings (valence, arousal and intensity). To assess whether the distributions of these ratings differed across emotion regulation conditions within each label group, the ratings were first plotted separately by condition and then statistically compared using energy distance tests [61] (a non-parametric test of pairwise distances). All energy distance tests were evaluated using 10 000 permutations and corrected for multiple comparisons using the Benjamini–Hochberg procedure. More specifically, the distributions of these ratings were then analysed for each of the dimensions (valence, arousal and intensity) by comparing the variance of the distributions using Levene's tests and were similarly corrected for multiple comparisons [62]. Pre-processing and analyses of emotion labels were carried out in Python.

## 3. Results

### (a) Reported emotional experience

#### (i) Main effect of video type

We first demonstrated that videos characterized *a priori* as negative were rated by participants as more negatively valenced (*t*(494) = 36.90, *SE* = 1.42, *p* < 0.001, *d* = 2.58), more arousing (*t*(494) = 18.02, *SE* = 1.70, *p* < 0.001, *d* = 1.31) and more intense (*t*(494) = 15.42, *SE* = 1.69, *p* < 0.001, *d* = 1.00), compared with neutral videos.

#### (ii) Main effect of emotion regulation condition

Comparison of the different emotion regulation conditions (watch, distance, immerse) on self-reported emotion experience revealed no main effect for valence (*F*(2,493) = 0.20, *p*-adjusted = 0.817, *η*²_*p*_ = 0.001), yet there were significant main effects for arousal (*F*(2,493) = 3.56, *p*-adjusted = 0.044, *η*²_*p*_ = 0.014) and intensity (*F*(2,493) = 18.57, *p*-adjusted < 0.001, *η*²_*p*_ = 0.070). *Post hoc* Tukey-adjusted pairwise comparisons found that those in the immerse condition reported higher arousal than in the distance condition (*p*-adjusted = 0.022, *SE* = 2.70, *d* = 0.27) and higher intensity than both watch (*p*-adjusted = 0.007, *SE* = 2.36, *d* = 0.27) and distance (*p*-adjusted < 0.001, *SE* = 2.44, *d* = 0.56); distance also elicited lower intensity than watch (*p*-adjusted = 0.007, *SE* = 2.50, *d* = 0.29).

#### (iii) Effect of emotion regulation condition by video type

Significant interactions were found between emotion regulation condition and video type on valence (*F*(2,490) = 14.41, *p*-adjusted < 0.001, *η*²_*p*_ = 0.056), signifying that participants’ valence ratings varied across emotion regulation conditions in different ways for negative versus neutral videos. However, no significant interactions were found for arousal (*F*(2,490) = 2.06, *p*-adjusted = 0.192, *η*²_*p*_ = 0.008) and intensity ratings (*F*(2,490) = 1.06, *p*-adjusted = 0.349, *η*²_*p*_ = 0.004).

**Figure 1.**
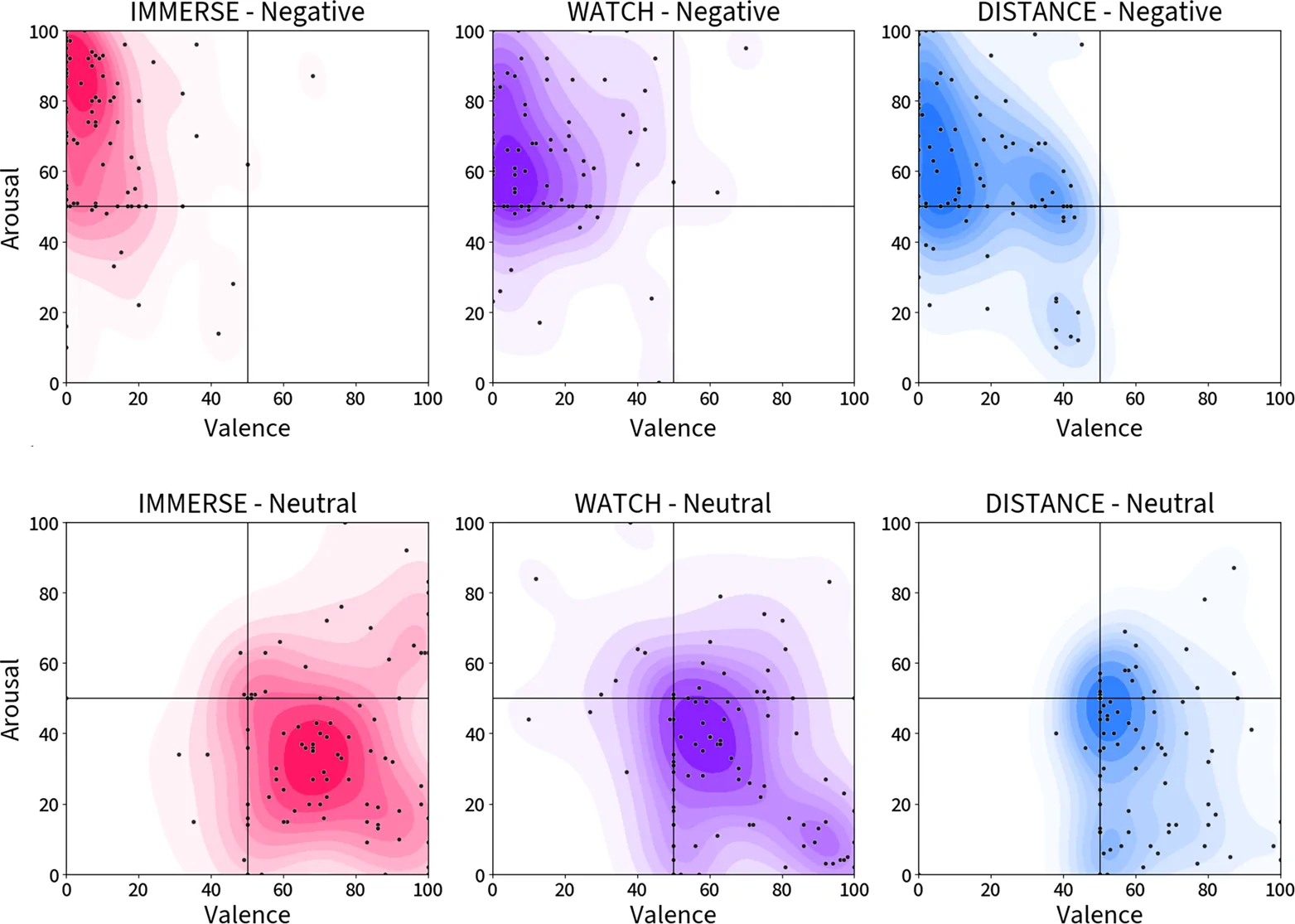
Distribution of participants’ ratings by video type and emotion regulation condition. Density plots illustrate the distribution of subjective ratings split by video type and emotion regulation condition. Scatterplots reflect the individual subjective ratings by participant.

For negative videos, there was a main effect of emotion regulation condition on valence ratings (*F*(2,223) = 10.08, *p*-adjusted < 0.001, *η*²_*p*_ = 0.083). Immersing led to more negative valence ratings than distancing (*t*(223) = 4.35, *SE* = 1.53, *p*-adjusted < 0.001, *d* = 0.42) and watching (*t*(223) = 2.60, *SE* = 1.59, *p*-adjusted = 0.027, *d* = 0.26). There was also a main effect for neutral videos, (*F*(2,223) = 9.70, *p*-adjusted < 0.001, *η*²_*p*_ = 0.082); however, the opposite effect was found: immersing yielded more positive valence ratings than distancing (*t*(223) = 4.40, *SE* = 2.48, *p*-adjusted < 0.001, *d* = 0.55) and watching (*t*(223) = 2.56, *SE* = 2.68, *p*-adjusted = 0.030, *d* = 0.35; see figure 1).

Together, these observations quantify the impact of emotional regulation strategies on experiential response, with heightened arousal and intensity through immersion compared with distancing for both negative and neutral videos. By contrast, immersion in negative videos resulted in more negative valence but was made more positive through immersion in neutral stimuli.

#### (iv) Emotion labels

Participants generated more labels after negative than neutral videos (Mann–Whitney *U* = 46 245, *p* < 0.001) and when immersing compared with distancing after correcting for multiple comparisons using the Benjamini–Hochberg procedure (*t*(358) = 3.03, *p-*adjusted = 0.008, *d* = 0.32). After pre-processing, the final label groupings were ‘disgust,’ ‘fear,’ ‘sadness,’ ‘anxiety’ and ‘anger’ for negative videos, and ‘calm,’ ‘happy’ and ‘neutral’ for neutral videos.

**Figure 2.**
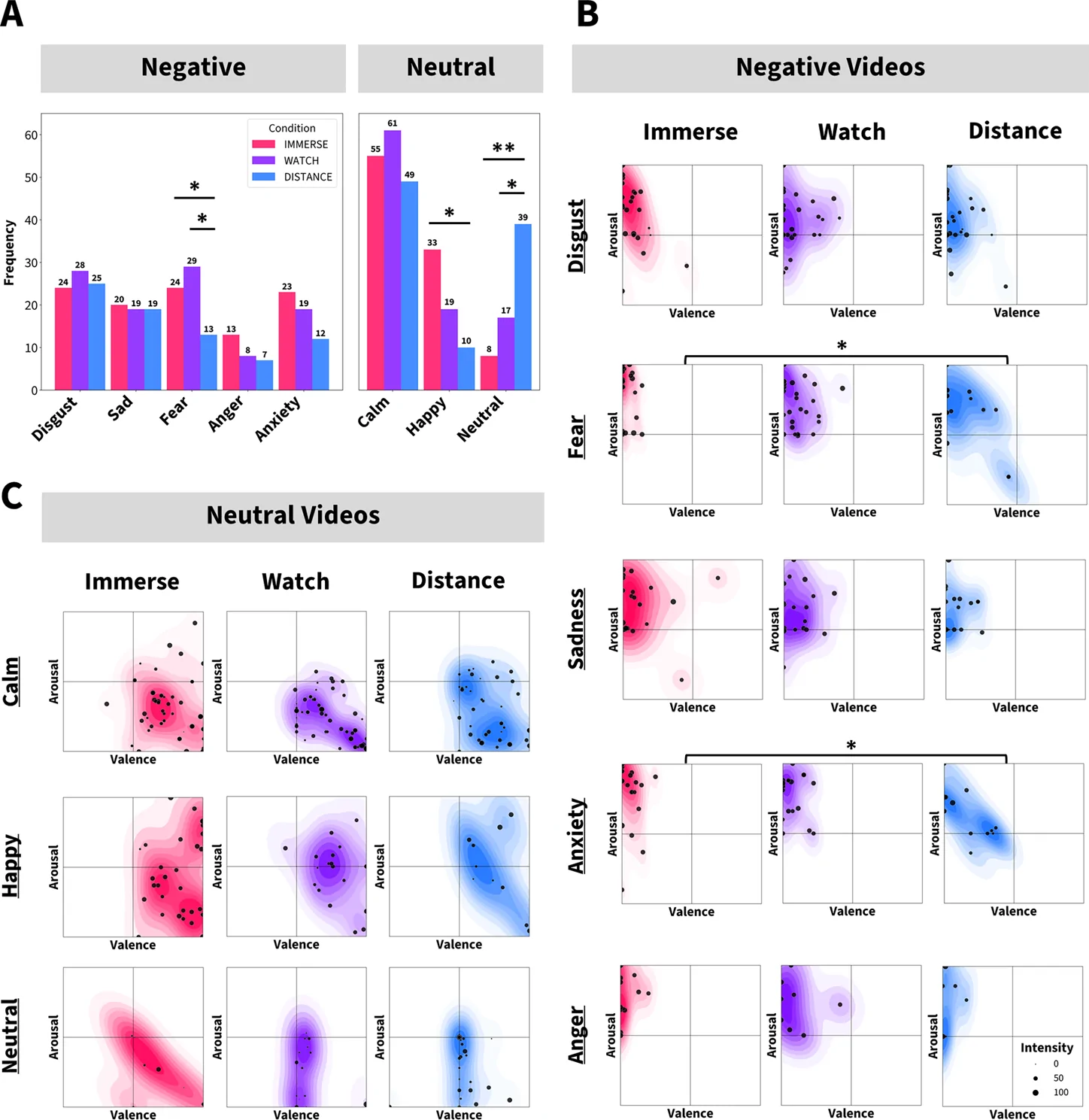
Distributions of emotion labels in negative and neutral videos. (A) Frequency histograms of all emotion label groupings by emotion regulation condition and by video valence. Each individual frequency refers to an instance of the specified emotion label generated in the specified emotion regulation condition. (B) The distribution of labels for each negative emotion label grouping on the affect grid. Distributions are split by column, based on the emotion regulation condition of the block in which the labels were generated. (C) The comparable distribution of labels for each neutral emotion label grouping on the affect grid. * *p*-adjusted < 0.05, *** *p*-adjusted < 0.001.

The frequency of labels was found to differ between conditions for labels corresponding to ‘fear’ (*χ*^2^(2) = 9.59, *p* = 0.008) for negative videos, and ‘happy’ (*χ*^2^(2) = 10.14, *p* = 0.006) and ‘neutral’ (*χ*^2^(2) = 17.45, *p* < 0.001) for neutral videos. *Post hoc* pairwise comparisons with Benjamini–Hochberg correction applied found that self-distancing reduced labels for ‘fear’ for negative videos compared with the baseline watch condition (*p*-adjusted = 0.020), along with the immerse condition (*p*-adjusted = 0.028). Self-distancing increased ‘neutral’ labels for neutral videos compared with watching (*p*-adjusted = 0.031) and when compared with immersing (*p*-adjusted < 0.001). Immersion reduced ‘neutral’ labels for neutral videos compared with watching (*p*-adjusted = 0.033) (figure 2A).

Results from the free generation of emotion labels linked to their corresponding self-reported valence, arousal and intensity ratings revealed how emotions may be organized in the three-dimensional valence–arousal–intensity space. Label distributions corresponded to distinct clusters when examined separately for negative and neutral videos (figure 2B,C). For negative videos, emotional regulation conditions significantly shifted ratings along valence–arousal–intensity dimensions for ‘fear’ (immerse versus distance: *E* = 12.57, *p-*adjusted = 0.047) and ‘anxiety’ (immerse versus distance: *E* = 14.89, *p*-adjusted = 0.047) label groups. Further analyses revealed differences in the effect of different emotion regulation conditions only in the valence dimension after Benjamini–Hochberg correction, where differences were found for ‘fear’ (immerse versus distance: *F*(1,35) = 9.94, *p*-adjusted = 0.040) and ‘anxiety’ (immerse versus distance: *F*(1,32) = 16.28, *p*-adjusted = 0.007) label groups. Non-significant results are detailed in tables S2–S9 in the supplementary material.

Together, these observations suggest an effect of emotion regulation, with reductions in emotion labels associated with fear when distancing compared with the baseline watch condition. Distancing and immersing resulted in significantly different organizations of ‘fear’ and ‘anxiety’ labels in the three-dimensional valence–arousal–intensity space. This was seen more specifically in the valence dimension, where distancing resulted in less negative ratings for ‘fear’ and ‘anxiety’ emotion labels compared with immersing. These results suggest that fear- and anxiety-based emotions may be particularly responsive to distancing strategies.

### (b) Physiology

#### (i) Main effect of video type

Across all participants, mean HR was significantly lower when viewing negative videos compared with neutral videos (*t*(494) = 5.23, *SE* = 0.27, *p*-adjusted < 0.001, *d* = 0.11). Nevertheless, negative videos were associated with a faster mean respiration rate (*t*(494) = 2.96, *SE* = 0.17, *p*-adjusted = 0.013, *d* = 0.15). RSA amplitude was also elevated (*t*(494) = 2.69, *SE* = 0.11, *p*-adjusted = 0.020, *d* = 0.20) for negative relative to neutral videos. However, other (time- and frequency-dependent) measures of HRV showed no differential effects between negative and neutral videos (RMSSD: *t*(494) = 1.22, *SE* = 3.61, *p*-adjusted = 0.449, *d* = 0.04; HF–HRV: *t*(494) = 0.06, *SE* = 0.003, *p*-adjusted = 0.956, *d* = 0.004).

#### (ii) Main effect of emotion regulation condition

When comparing the effects of emotion regulation condition on physiology, no significant main effects were found when collapsing across video type (*p* > 0.095).

#### (iii) Effect of emotion regulation condition split by video type

No significant interactions between emotion regulation condition and video type on physiology were found, with no observed differences in physiological indices between emotion regulation conditions separately in negative and neutral videos after correcting for multiple comparisons.

**Figure 3.**
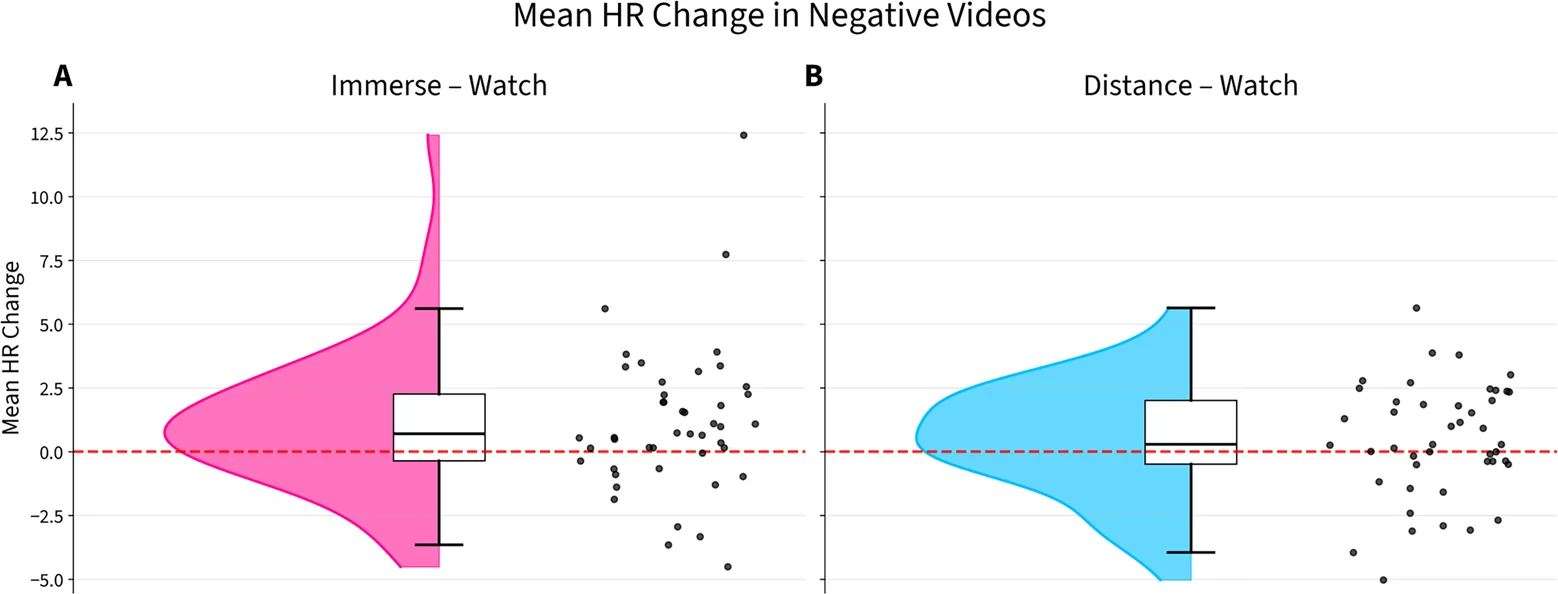
Violin and scatterplot of the difference in mean HR from watch condition for negative videos. (A) Difference in mean HR (immerse–watch) for each participant, where values above 0 (red line) indicate a higher mean HR when immersing compared with simply watching the negative videos. (B) Difference in mean HR (distance–watch) for each participant, where values above 0 (red line) indicate an increase in mean HR when distancing compared with simply watching the negative videos.

However, uncorrected significant interactions were found. First, for negative videos, there was a main effect of emotion regulation condition (*F*(2,223) = 3.75, *p* = 0.025, *η*²_*p*_ = 0.033), where the mean HR was higher when immersing than watching (*t*(223) = 2.72, *SE* = 0.41, *p* = 0.007, *d* = 0.09). There was no significant difference in mean HR when distancing compared with watching (*t*(484) = 1.09, *SE* = 0.41, *p* = 0.275, *d* = 0.05) (figure 3). No main effect was found for neutral videos (*F*(2,223) = 0.33, *p* = 0.719, *η*²_*p*_ = 0.003).

There were also no overall observed differences in RSA nor HF-HRV (proximate indices of cardiorespiratory coupling) between emotion regulation conditions for negative videos (RSA: *F*(2,223) = 0.35, *p* = 0.702, *η*²_*p*_ = 0.003; HF–HRV: *F*(2,223) = 1.01, *p* = 0.367, *η*²_*p*_ = 0.009). However, for negative videos, the time-dependent measure of HRV (RMSSD) showed an effect of emotion regulation condition (*F*(2,223) = 3.11, *p* = 0.047, *η*²_*p*_ = 0.027) and it was lower when immersing in negative emotional content compared with the watching condition (*t*(223) = 2.47, *SE* = 3.24, *p* = 0.038, *d* = 0.09). However, there was no significant difference when distancing was compared with watching (*t*(484) = 1.54, *SE* = 3.24, *p* = 0.275, *d* = 0.06). No main effect was observed for neutral videos (RSA: *F*(2,223) = 0.60, *p* = 0.549, *η*²_*p*_ = 0.005; HF–HRV: *F*(2,223) = 0.57, *p* = 0.564, *η*²_*p*_ = 0.005; RMSSD: *F*(2,223) = 1.42, *p* = 0.244, *η*²_*p*_ = 0.013).

Despite the observed faster breathing in response to negative videos, mean respiration rate, RRV or I/E ratio were not observed to change as a function of emotion regulation condition for either negative (mean respiration rate: *F*(2,223) = 1.00, *p* = 0.371, *η*²_*p*_ = 0.009; time-dependent measure of RRV: *F*(2,223) = 0.87, *p* = 0.422, *η*²_*p*_ = 0.008; frequency-dependent measure of RRV: *F*(2,223) = 0.60, *p* = 0.548, *η*²_*p*_ = 0.005; I/E ratio: *F*(2,223) = 1.08, *p* = 0.343, *η*²_*p*_ = 0.010) or neutral videos (mean respiration rate: *F*(2,223) = 1.12, *p* = 0.327, *η*²_*p*_ = 0.010; time-dependent measure of RRV: *F*(2,223) = 2.06, *p* = 0.130, *η*²_*p*_ = 0.018; frequency-dependent measure of RRV: *F*(2,223) = 0.82, *p* = 0.442, *η*²_*p*_ = 0.007; I/E ratio: *F*(2,223) = 0.01, *p* = 0.991, *η*²_*p*_ = 0.0001).

Absolute change scores, calculated to assess the impact of emotion regulation on cardiac reactivity irrespective of direction, revealed significant absolute changes in mean HR across all videos (*t*(179) = 14.06, *SE* = 2.16, *p* < 0.001, *d* = 1.05) and during self-distancing (*t*(89) = 12.31, *SE* = 2.05, *p*-adjusted < 0.001, *d* = 1.30) and self-immersion (*t*(89) = 8.78, *SE* = 2.28, *p*-adjusted < 0.001, *d* = 0.92) compared with watching.

There was no significant interaction between video type and comparison condition (*F*(1,132) = 0.22, *p* = 0.643, *η*²_*p*_ = 0.002): similar patterns of absolute change in mean HR were found in both negative (distance versus watch: *t*(44) = 8.49, *SE* = 1.77, *p*-adjusted < 0.001, *d* = 1.27, immerse versus watch: *t*(44) = 6.33, *SE* = 2.13, *p*-adjusted < 0.001, *d* = 0.94) and neutral videos (distance versus watch: *t*(44) = 9.13, *SE* = 2.33, *p*-adjusted < 0.001, *d* = 1.36, immerse versus watch: *t*(44) = 6.10, *SE* = 2.42, *p*-adjusted < 0.001, *d* = 0.91).

## 4. Discussion

In the current study, we investigated self-reported (experiential) and physiological consequences of emotion regulation strategies. Using two emotion regulation strategies, heightening emotion through immersion or attenuating emotion through distancing, we measured self-reported emotional arousal, valence and intensity and self-generated emotion terms. In parallel, we quantified the effect of emotion regulation strategies on autonomic physiology, focusing on respiratory and cardiac measures. Finally, we investigated the link between the two: how subjective emotional experience relates to autonomic physiology, and how emotion regulation strategies influence this relationship.

We demonstrated that videos selected *a priori* to be emotionally negative successfully elicited corresponding emotional ratings, relative to neutral videos, as demonstrated in both self-report and autonomic measures. We found relationships between emotion regulation strategy and subjective measures of emotion with negative videos, with distancing associated with lower arousal and intensity ratings, and immersing with more negative valence and higher arousal ratings (even compared with simply watching). Freely generated emotion labels relating to fear and anxiety were more likely to be reduced through self-distancing, whereas no significant effect of self-distancing was observed on labels corresponding to disgust, sadness and anger. Physiologically, viewing negative videos resulted in a decrease in HR, and increases in respiration rate and RSA. Immersing in negative videos may also result in an increase in HR and a decrease in the time-dependent measure of HRV (RMSSD) compared with watching, although it should be noted that these findings did not survive corrections for multiple comparisons. Together, our findings suggest that emotion regulation strategies alter both subjective experiential and physiological indices of emotion, and that certain emotions, such as fear and anxiety, may be more sensitive to the strategy of distancing. In addition, the association between physiological reactivity and subjective emotional experience appears to depend on the type of emotional stimulus and the emotion regulation strategy applied.

### (a) Subjective emotional experience

We found that, as expected, viewing negative videos resulted in feelings of more negative valence and higher arousal as compared with neutral videos. Self-distancing resulted in a general reduction in arousal and intensity across both negative and neutral videos, while immersing elicited a general increase in arousal and intensity across both emotional and neutral videos. By contrast, immersing in negative videos elicited a more negative valence, while immersing in neutral videos resulted in a more positive valence. This pattern is consistent with the interpretation that immersion amplifies subjective emotional experience, as reflected in the more negative valence ratings for negative videos and more positive valence ratings for neutral stimuli. Indeed, in the watch condition, neutral videos were assessed on average as having mild positive valence, an effect that is amplified with ‘immersion’ in these videos. This could be owing to increased self-relevance to and elaboration of emotional information, by which the pre-dominant affective tone of a stimulus may be amplified. This is consistent with evidence that increased self-referential processing and mental imagery may amplify emotional experience by encouraging individuals to mentally simulate events as personally or socially relevant (see review by Holmes & Mathews [63]).

An alternative account for the effect of immersion on subjective emotional experience is that immersion may alter appraisal processes in different directions depending on individuals’ baseline affective valence. Rather than amplifying the pre-dominant affective tone of a stimulus, an individual's baseline affect itself would be amplified with immersion instructions. This effect would be consistent with the view that baseline affect drives the saliency of features under immersion instructions, where a more positive baseline affect would make subtle positive cues more salient, whereas negative videos may be judged as more negative because aversive features receive greater attention with immersion [64,65]. However, this latter interpretation is unlikely. Participants were not informed about the valence of upcoming videos, thus anticipatory affect was unlikely to systematically shape appraisal. Blocks also alternated between negative and neutral videos, thus any carry-over effects from the preceding block would tend to produce a baseline affect that is incongruent with the valence of the upcoming blocks. For example, a negative block always follows a neutral block, and *vice versa*. This pattern would thus predict effects that are opposite to those that were observed here. In addition, this cannot be a trait-level effect. If the effects of immersion reflected amplification of an individual's baseline affect, then individuals with more positive (or negative) baseline affect should show consistent shifts in valence ratings across all trials. That is to say, if an individual has a more positive baseline affect, they may view subtle positive cues as more salient across both negative and neutral videos, and *vice versa*. Therefore, a stable baseline affect should influence immersion-related appraisals in a consistent direction across all stimuli rather than producing more negative valence ratings for negative videos and more positive valence ratings for neutral videos.

These observations reinforce previous literature on the effects of self-distancing, where it is well established that it reduces negative affect in response to emotional stimuli [26,27,32]. Even though less research has investigated self-immersion, our findings support previous research where amplification of subjective emotional experience is observed in response to immersion in emotional stimuli [30]. Published studies comparing self-distancing with self-immersing report similar results, although participants reflect retrospectively [35] or prospectively [36] on negative events and report whether they were reflecting from a self-distanced or self-immersed perspective. Our results thus add to evidence regarding the impact of concurrent, retrospective and prospective self-distancing during negative emotional events. A two-dimensional affect structure of valence and arousal is widely applied in studies of emotion. However, all aspects of emotion experience may not be properly captured within such a two-dimensional model—for example, how much a participant ‘feels’ an emotion. The addition of an intensity dimension attempts to address this, and indeed our study was able to quantify changes elicited by distinct types of emotion regulation strategies. Consistent with previous work, self-distancing attenuated subjective emotional intensity [66–68] compared with self-immersing. However, it is also possible that the reduction in emotional intensity observed during distancing reflects a deployment of mechanisms other than psychological distance alone, such as, suppression (or inhibition) of emotional reactions to the videos [69].

By allowing participants to label their emotions freely, rather than selecting from a pre-defined list, we captured a broader range of subjective emotional experiences in response to the video stimuli. Examination of the most frequently reported emotion labels on the affect grid revealed distinct clusters for labels associated with negative and neutral videos, with observable shifts across emotion regulation conditions. Self-distancing reduced the frequency of labels pertaining to ‘fear’ compared with just watching and self-immersing in negative videos. Self-distancing also influenced the emotional experience of ‘fear’ labels compared with self-immersing, resulting in a more heterogeneous emotional experience among participants, with some participants rating their emotion as less negatively valenced, less arousing and less intense. Specifically, our findings suggest that emotion regulation strategies particularly influence subjective valence for the negative emotion labels ‘fear’ and ‘anxiety’, but they have less impact on rated arousal and intensity. However, to maximize statistical power, analyses focused on the most frequently reported emotions, with semantically similar categories combined after confirming no significant differences in their affect grid distributions. This approach presents limitations because semantically similar emotions may nonetheless represent distinct emotional states, potentially biasing the findings toward more ‘basic’ emotion labels. This grouping approach may obscure meaningful distinctions between semantically related emotional states (e.g. fear versus panic, sadness versus grief) in how they respond to self-distancing, which could potentially explain the more heterogeneous nature of emotional experience among participants. As such, results presented here should be interpreted within the context of the grouping approach taken. Disentangling such differences would require larger samples to determine whether these emotions are meaningfully distinct from one another, and to provide sufficient power to detect more subtle differences in subjective arousal that may not have been shown in our results. Nevertheless, this strategy yielded interesting insights, where distancing significantly reduced the number of words generated that mapped onto fear. This finding has implications for cognitive therapeutic strategies where techniques reliant on principles of distancing and decentring may be particularly effective for both anxiety- and fear-based symptomatology.

### (b) Physiology

We found that, at the group level, mean HR was lower during negative videos, while mean respiration rate and RSA were higher compared with neutral videos. This bradycardia response to negative relative to neutral videos is aligned with other studies’ findings looking at negative emotions like disgust [70] and sadness [71,72] as induced from viewing video clips, as well as from studies employing negatively valenced pictures from the International Affective Picture System [73,74]. This pattern corresponds to parasympathetic dominance of the nervous system, supporting previous research detailing parasympathetic activation during the processing of disgust videos [75–77]. This pattern has also been found with viewing sad videos, with bradycardia and increased RSA being reported [78], although the bradycardia response as a reaction to sadness can be modulated by the context of the video stimuli [79]. Fear and anxiety can also elicit lower HR, particularly in the case of phobia and anxious anticipation [80–84], which have been associated with a vagally mediated defence response to highly arousing negative stimuli [85]. HR deceleration following stimulus presentation has also been linked with the orienting response [86,87], which is thought to involve parasympathetic or vagal inhibitory mechanisms supporting attentional engagement with salient stimuli [88]. Speculatively, it is possible that our blocked design, which meant that multiple negative videos were presented in succession, may have prolonged the orienting response throughout each block—an effect augmented for negatively blocked stimuli [89].

Moreover, more negative videos may have elicited states of spontaneous regulation [90], as indexed by a reduction in HR across all emotion regulation conditions, even in the watch condition. Thus, there may be individual differences in the direction of physiological change in response to negative videos—such as the nature of the emotions that they personally felt while watching them and spontaneous regulation—in addition to other factors, such as individual anticipatory responses to the videos. This could explain the failure to find any main effect of emotion regulation condition for other physiological indices, including mean respiration rate and the RSA measure of HRV, because different individuals may have had differing directions of physiological change as a result of individual differences. Supporting this interpretation, we found significant absolute changes in mean HR with emotion regulation conditions, irrespective of direction, for both self-distancing and self-immersing as compared with watching for both negative and neutral videos.

Together, these results indicate that the effects of emotion regulation on physiology are complex, with different individuals having differing directions of physiological change in response to emotion regulation. Analyses at the group level are not able to probe these subtle individual difference measures, and as such, a larger sample is required to map and identify certain clusters of individuals who may react in a certain way to emotion regulation. The relationship between subjective emotional experience and physiology should also be modelled to provide a more integrated understanding of emotion regulation processes. Owing to the lack of power from our final sample size coupled with substantial inter-individual variability in physiological responding, we could not reliably ascertain these effects. To help inform and power future studies, these results are reported in the supplementary material.

In addition, although we did not find a significant effect of medication and psychiatric status on our findings, the results here should still be interpreted in light of these factors because our inclusion criteria were designed to obtain a more naturalistic sample that is representative of the broad affective range across the general population. The number of participants self-reporting a diagnosis of affective and neurodevelopmental conditions was low, and thus unlikely to have a significant impact. However, we were not sufficiently powered to look at this in detail for each condition. In addition, breathing depth/tidal volume (which influence RSA and I/E ratio) were not assessed, which presents as a limitation given that the different emotion regulation conditions may implicitly induce differences in breathing patterns [91,92]. Moreover, whereas the previously mentioned studies examined discrete negative emotions, participants in the present study were permitted to freely label their subjective emotional experiences. Given the limited sample size overall for each reported emotion, it was not possible to examine physiological reactivity separately for each emotion across regulation conditions. As certain emotions may be associated with distinct physiological profiles [10], future studies with larger samples will be necessary to determine whether different negative emotions are characterized by differential physiological responses under varying emotion regulation strategies.

### (c) Future directions and conclusions

Future research should further examine the role of individual differences, such as emotional granularity, which is the capacity to differentiate and label one's emotional states, given prior evidence linking higher negative emotional granularity to more effective emotion regulation [93]. In addition, individual differences particularly surrounding autonomic responses to different emotions and differing impact of regulation strategies on the direction of autonomic change should be examined. The use of time-series approaches could offer valuable insights into the temporal dynamics of these processes. For example, examining the coupling between cardiac and respiratory activity across different video types and emotion regulation conditions may reveal more fine-grained mechanisms underlying the coordination of physiological systems during emotion regulation. Further research should also be carried out to ascertain the potential influence (or lack thereof) of respiration on the effects of emotion regulation on cardiovascular physiology, given its clinical implications, with methods such as deep breathing or paced breathing being used to help regulate emotion.

In conclusion, the present study provides evidence that emotion regulation strategies like self-distancing influence subjective emotional experiences, with complex effects on physiological activity. These findings extend prior work by addressing gaps in the literature, including the limited evidence on the psychophysiological effects of emotion regulation strategies, as well as the subjective evaluation of freely-generated emotion labels as a function of emotion regulation strategy. We present initial evidence that fear- and anxiety-based emotions may be more sensitive to distancing strategies.

## Supplementary Material



## Data Availability

The data and code can be accessed in the Electronic Supplementary Material, or at https://github.com/TheEmBodyStudy/VideoTask-Validation [94]. Supplementary material is available online [95].

## Theme

One contribution of 19 to a theme issue ‘Mechanisms, development, phylogeny and functions of emotional expressions’.
