# Supplementary material for: The impact of emotion regulation strategies on experiential and physiological responses to negative emotional videos
Source: Philos Trans R Soc Lond B Biol Sci. 2026 Sep 24;381(1959):20250138. doi: 10.1098/rstb.2025.0138 (PMC13601890; doi:10.1098/rstb.2025.0138)
Supplement: Supplementary file 1 [file rstb.20250138.zip › rstb.20250138/rstb20250138_si_001.docx]

**Supplementary Material**

**Relationship between self-reported emotional experience and physiology**

***Valence ratings***

Our findings above show no significant differences in physiological responses to video stimuli categorised a priori as negative and neutral, although uncorrected results indicate few, sometimes counterintuitive, differences in the physiological responses to video stimuli categorised a priori as negative and neutral. However, when more fine-grained self-reported affective ratings were considered, we observed that higher mean HR predicted more negative valence ratings *(t*(484) = 1.97, *SE* = 0.15, *p* = .049). Significant three-way interactions were also found between: (1) respiration rate, video type, and emotion regulation condition (*t*(484) = 2.37, *SE* = 0.84, *p* = .018), and (2) the time-dependent measure of RRV, video type, and emotion regulation condition (*t*(484) = 2.57, *SE* = 0.003, *p* = .011). Simple slope analyses found a significant negative association between RRV (RMSSD) and valence in the neutral distance blocks (*t*(484) = 2.03, *SE* = 0.002, *p* = .043): More regular breathing when distancing led to neutral videos being rated as more positively valenced. However, post-hoc pairwise comparisons did not yield any significant results (all *p*s > .159).

***Arousal ratings***

A significant interaction between mean HR and emotion regulation condition was observed, (*t*(484) = 2.28, *SE* = 16.59, *p* = .023): Higher heart rate predicted higher self-rated arousal during the Immerse condition, in contrast to Watch and Distance conditions. Simple slopes analyses found that these were not significant (*t*(484) = 0.86, *SE* = 0.15, *p* = .393). Post-hoc pairwise comparisons showed the Immerse association differed significantly from Distance (*t*(484) = 2.68, *SE* = 0.14, *p*-adjusted = .021) and Watch (*t*(484) = 2.51, *SE* = 0.14, *p*-adjusted = .034). Another significant interaction between I/E ratio and video type was also observed (*t*(484) = 2.02, *SE* = 19.34, *p* = .044): Longer expiration reduced evoked arousal to negative videos compared to neutral videos. Simple slope analyses found that this negative association between I/E ratio and arousal in negative videos was significant (*t*(484) = 2.26, *SE* = 6.49, *p* = .024), and post-hoc pairwise comparisons found that the negative association differed significantly from neutral videos (*t*(484) = 2.43, *SE* = 10.60, *p*-adjusted = .015).

***Intensity ratings***

A significant three-way interaction between RSA, video type, and emotion regulation condition was observed (*t*(484) = 2.09, *SE* = 2.43, *p* = .037): Interestingly, higher RSA predicted higher self-ratings of emotional intensity during the Immerse condition for negative videos, but lower self-ratings for neutral videos. Simple slope analyses found that the negative association between RSA and intensity was significant (*t*(484) = 2.03, *SE* = 0.99, *p* = .043), but post-hoc pairwise comparisons did not find any significant results (all *p*s > .174).

Notably, the interaction between the time-dependent measure of HRV (RMSSD) and emotion regulation condition was significant (*t*(484) = 2.66, *SE* = 0.04, *p* = .008): Higher HRV (RMSSD) predicted higher intensity during Distance and Immerse condition, and a lower intensity during the Watch condition. Simple slopes showed a positive association during Distance (*t*(484) = 2.16, *SE* = 0.02, *p* = .031), where an increased HRV (RMSSD) was associated with increased intensity. Post-hoc pairwise comparisons indicated a significant difference between Watch and Distance (*t*(484) = 2.51, *SE* = 0.02, *p*-adjusted = .033), while other comparisons did not find significant differences (Watch-Immerse: *t*(484) = -1.66, *SE* = 0.02, *p* = .222; Distance-Immerse: *t*(484) = 1.10, *SE* = 0.02, *p* = .515).

**Figure 1**

*Task overview*

**Table 1**

*Breakdown of Label Groupings*

| Label group | Video Type | Frequency | Contains |
| --- | --- | --- | --- |
| Disgust | Negative | 75 | Disgust |
| Fear | Negative | 66 | Scare, Fear |
| Sad | Negative | 58 | Sad |
| Anxiety | Negative | 52 | Worry, Anxiou, Nervou |
| Anger | Negative | 28 | Anger |
| Calm | Neutral | 161 | Calm, Relax, Peac |
| Happy | Neutral | 61 | Happy, Joy, Pleasant |
| Neutral | Neutral | 58 | Noth, Bore, Indiffer, Neutral |

**Table 2**

*Energy Distance Tests of Affect Grid Distributions (Negative Videos)*

| Label group and comparison | *E*-statistic | *p*-uncorrected | *p*-adjusted (FDR) |
| --- | --- | --- | --- |
| Disgust |  |  |  |
| Watch vs. Immerse | -0.68 | .523 | .698 |
| Watch vs. Distance | -1.37 | .999 | .999 |
| Immerse vs. Distance | -0.42 | .407 | .698 |
| Fear |  |  |  |
| Watch vs. Immerse | 3.04 | .045 | .180 |
| Watch vs. Distance | 2.08 | .121 | .365 |
| Immerse vs. Distance | 12.57 | .004 | .047 |
| Sadness |  |  |  |
| Watch vs. Immerse | -0.91 | .626 | .791 |
| Watch vs. Distance | -1.97 | .999 | .999 |
| Immerse vs. Distance | -0.52 | .439 | .698 |
| Anxiety |  |  |  |
| Watch vs. Immerse | -0.30 | .390 | .698 |
| Watch vs. Distance | 8.72 | .026 | .123 |
| Immerse vs. Distance | 14.89 | .004 | .047 |
| Anger |  |  |  |
| Watch vs. Immerse | -1.39 | .514 | .698 |
| Watch vs. Distance | -1.16 | .479 | .698 |
| Immerse vs. Distance | -0.50 | .425 | .698 |

**Table 3**

*Energy Distance Tests of Affect Grid Distributions (Neutral Videos)*

| Label group and comparison | *E*-statistic | *p*-uncorrected | *p*-adjusted (FDR) |
| --- | --- | --- | --- |
| Calm |  |  |  |
| Watch vs. Immerse | 0.51 | .159 | .382 |
| Watch vs. Distance | 0.73 | .138 | .369 |
| Immerse vs. Distance | 2.57 | .022 | .123 |
| Happy |  |  |  |
| Watch vs. Immerse | 6.54 | .016 | .123 |
| Watch vs. Distance | -2.84 | .999 | .999 |
| Immerse vs. Distance | 5.64 | .070 | .240 |
| Neutral |  |  |  |
| Watch vs. Immerse | -4.83 | .999 | .999 |
| Watch vs. Distance | -0.79 | .486 | .698 |
| Immerse vs. Distance | -2.57 | .766 | .919 |

**Table 4**

*Variance of Valence Ratings by Label Group for Negative Videos*

| Label group and comparison | *F*-statistic | *df*1 | *df2* | *p*-uncorrected | *p*-adjusted (FDR) |
| --- | --- | --- | --- | --- | --- |
| Disgust |  |  |  |  |  |
| Watch vs. Immerse | 0.80 | 1 | 48 | .375 | .820 |
| Watch vs. Distance | 0.38 | 1 | 49 | .540 | .845 |
| Immerse vs. Distance | 0.05 | 1 | 47 | .823 | .898 |
| Fear |  |  |  |  |  |
| Watch vs. Immerse | 6.28 | 1 | 51 | .015 | .093 |
| Watch vs. Distance | 3.06 | 1 | 40 | .088 | .352 |
| Immerse vs. Distance | 9.94 | 1 | 35 | .003 | .040 |
| Sadness |  |  |  |  |  |
| Watch vs. Immerse | 0.32 | 1 | 37 | .574 | .846 |
| Watch vs. Distance | 0.41 | 1 | 36 | .528 | .845 |
| Immerse vs. Distance | 0.90 | 1 | 37 | .350 | .820 |
| Anxiety |  |  |  |  |  |
| Watch vs. Immerse | 0.52 | 1 | 38 | .476 | .845 |
| Watch vs. Distance | 8.20 | 1 | 28 | .008 | .063 |
| Immerse vs. Distance | 16.28 | 1 | 32 | <.001 | .008 |
| Anger |  |  |  |  |  |
| Watch vs. Immerse | 1.54 | 1 | 19 | .229 | .688 |
| Watch vs. Distance | 0.91 | 1 | 13 | .358 | .820 |
| Immerse vs. Distance | 0.01 | 1 | 18 | .916 | .956 |

**Table 5**

*Variance of Arousal Ratings by Label Group for Negative Videos*

| Label group and comparison | *F*-statistic | *df*1 | *df2* | *p*-uncorrected | *p*-adjusted (FDR) |
| --- | --- | --- | --- | --- | --- |
| Disgust |  |  |  |  |  |
| Watch vs. Immerse | 0.23 | 1 | 48 | .633 | .820 |
| Watch vs. Distance | 0.06 | 1 | 49 | .806 | .885 |
| Immerse vs. Distance | 0.06 | 1 | 47 | .811 | .885 |
| Fear |  |  |  |  |  |
| Watch vs. Immerse | 0.12 | 1 | 51 | .734 | .975 |
| Watch vs. Distance | 0.08 | 1 | 40 | .781 | .885 |
| Immerse vs. Distance | 0.17 | 1 | 35 | .684 | .885 |
| Sadness |  |  |  |  |  |
| Watch vs. Immerse | 0.07 | 1 | 37 | .790 | .885 |
| Watch vs. Distance | 0.67 | 1 | 36 | .418 | .885 |
| Immerse vs. Distance | 1.10 | 1 | 37 | .301 | .885 |
| Anxiety |  |  |  |  |  |
| Watch vs. Immerse | 0.14 | 1 | 38 | .710 | .885 |
| Watch vs. Distance | 0.25 | 1 | 28 | .619 | .885 |
| Immerse vs. Distance | 0.001 | 1 | 32 | .975 | .975 |
| Anger |  |  |  |  |  |
| Watch vs. Immerse | 0.005 | 1 | 19 | .945 | .975 |
| Watch vs. Distance | 0.64 | 1 | 13 | .438 | .885 |
| Immerse vs. Distance | 0.75 | 1 | 18 | .397 | .956 |

**Table 6**

*Variance of Intensity Ratings by Label Group for Negative Videos*

| Label group and comparison | *F*-statistic | *df*1 | *df2* | *p*-uncorrected | *p*-adjusted (FDR) |
| --- | --- | --- | --- | --- | --- |
| Disgust |  |  |  |  |  |
| Watch vs. Immerse | 0.03 | 1 | 48 | .860 | .898 |
| Watch vs. Distance | 3.66 | 1 | 49 | .061 | .246 |
| Immerse vs. Distance | 2.86 | 1 | 47 | .097 | .291 |
| Fear |  |  |  |  |  |
| Watch vs. Immerse | 0.18 | 1 | 51 | .675 | .884 |
| Watch vs. Distance | 0.06 | 1 | 40 | .810 | .884 |
| Immerse vs. Distance | 0.22 | 1 | 35 | .638 | .884 |
| Sadness |  |  |  |  |  |
| Watch vs. Immerse | 0.78 | 1 | 37 | .381 | .752 |
| Watch vs. Distance | 2.06 | 1 | 36 | .159 | .425 |
| Immerse vs. Distance | 0.53 | 1 | 37 | .471 | .754 |
| Anxiety |  |  |  |  |  |
| Watch vs. Immerse | 0.69 | 1 | 38 | .411 | .752 |
| Watch vs. Distance | 4.06 | 1 | 28 | .053 | .246 |
| Immerse vs. Distance | 7.9 | 1 | 32 | .008 | .101 |
| Anger |  |  |  |  |  |
| Watch vs. Immerse | 1.54 | 1 | 19 | .229 | .550 |
| Watch vs. Distance | 9.62 | 1 | 13 | .008 | .101 |
| Immerse vs. Distance | 3.13 | 1 | 18 | .094 | .291 |

**Table 7**

*Variance of Valence Ratings by Label Group for Neutral Videos*

| Label group and comparison | *F*-statistic | *df*1 | *df2* | *p*-uncorrected | *p*-adjusted (FDR) |
| --- | --- | --- | --- | --- | --- |
| Calm |  |  |  |  |  |
| Watch vs. Immerse | 0.22 | 1 | 113 | .637 | .637 |
| Watch vs. Distance | 0.62 | 1 | 104 | .433 | .845 |
| Immerse vs. Distance | 0.07 | 1 | 99 | .798 | .898 |
| Happy |  |  |  |  |  |
| Watch vs. Immerse | 0.28 | 1 | 49 | .599 | .845 |
| Watch vs. Distance | 0.12 | 1 | 27 | .736 | .898 |
| Immerse vs. Distance | 0.001 | 1 | 40 | .969 | .969 |
| Neutral |  |  |  |  |  |
| Watch vs. Immerse | 2.68 | 1 | 21 | .117 | .400 |
| Watch vs. Distance | 0.10 | 1 | 49 | .749 | .898 |
| Immerse vs. Distance | 4.11 | 1 | 40 | .049 | .237 |

**Table 8**

*Variance of Arousal Ratings by Label Group for Neutral Videos*

| Label group and comparison | *F*-statistic | *df*1 | *df2* | *p*-uncorrected | *p*-adjusted (FDR) |
| --- | --- | --- | --- | --- | --- |
| Calm |  |  |  |  |  |
| Watch vs. Immerse | 2.24 | 1 | 113 | .137 | .885 |
| Watch vs. Distance | 7.53 | 1 | 104 | .007 | .171 |
| Immerse vs. Distance | 0.63 | 1 | 99 | .429 | .898 |
| Happy |  |  |  |  |  |
| Watch vs. Immerse | 3.61 | 1 | 49 | .063 | .759 |
| Watch vs. Distance | 1.2 | 1 | 27 | .283 | .845 |
| Immerse vs. Distance | 0.13 | 1 | 40 | .723 | .885 |
| Neutral |  |  |  |  |  |
| Watch vs. Immerse | 0.32 | 1 | 21 | .576 | .885 |
| Watch vs. Distance | 0.11 | 1 | 49 | .740 | .885 |
| Immerse vs. Distance | 0.14 | 1 | 40 | .715 | .885 |

**Table 9**

*Variance of Intensity Ratings by Label Group for Neutral Videos*

| Label group and comparison | *F*-statistic | *df*1 | *df2* | *p*-uncorrected | *p*-adjusted (FDR) |
| --- | --- | --- | --- | --- | --- |
| Calm |  |  |  |  |  |
| Watch vs. Immerse | 0.21 | 1 | 113 | .648 | .752 |
| Watch vs. Distance | 0.21 | 1 | 104 | .648 | .752 |
| Immerse vs. Distance | 0.09 | 1 | 99 | .766 | .884 |
| Happy |  |  |  |  |  |
| Watch vs. Immerse | 0.01 | 1 | 49 | .911 | .911 |
| Watch vs. Distance | 3.98 | 1 | 27 | .056 | .246 |
| Immerse vs. Distance | 4.24 | 1 | 40 | .046 | .246 |
| Neutral |  |  |  |  |  |
| Watch vs. Immerse | 0.07 | 1 | 21 | .790 | .884 |
| Watch vs. Distance | 0.70 | 1 | 49 | .408 | .752 |
| Immerse vs. Distance | 0.08 | 1 | 40 | .775 | .884 |
